# Peritoneal hydatidosis: An exceptional case report

**DOI:** 10.1016/j.amsu.2022.104606

**Published:** 2022-09-10

**Authors:** Abdelilah El bakouri, Amine Fatine, Yassine Eddaoudi, Mounir Bouali, Khalid El Hattabi, Fatimazahra Bensardi, Abdelaziz Fadil

**Affiliations:** Visceral Surgical Emergency Department, Faculty of Medicine and Pharmacy, Universitary Hospital Center Ibn Rochd, Hassan II University, Casablanca, Morocco

**Keywords:** Hydatidosis, Exceptional hydatid cyst, Echinococcus granulosus, Treatment, Prognosis

## Abstract

**Introduction:**

Hydatidosis is a cosmopolitan parasitic disease which presents a real public health problem especially in endemic countries of which Morocco is part. The objective of the present work is to analyze the clinical, paraclinical, therapeutic, evolutionary and prognostic aspects of disseminated peritoneal hydatidosis with multiple localization.

Peritoneal hydatidosis represents the whole of the phenomena due to :

The seeding, essentially secondary, of the peritoneal serosa by Echinococcus Granulosus larvae, Peritoneal hydatidosis is characterized by its polymorphic symptomatology, and the diagnosis is based on a combination of epidemiological, clinical, biological and imaging findings.

**Materials and methods:**

We report a case of a particular form of peritoneal hydatidosis in the department of visceral surgery I of the ibn rochd hospital in casablanca.

**Results:**

Our patient was admitted for management of disseminated peritoneal hydatidosis. The clinical examination, apart from an epigastric crust, was unremarkable. The biological work-up showed a slightly disturbed liver balance and the hydatid serology was strongly positive. The preoperative diagnosis of HP was established by CT scan showing a supra- and sub-mesocolic peritoneal hydatidosis with a multi-cystic spleen and a liver with a type V segment V hydatid cyst measuring 4 cm by 6 cm.

The treatment consisted of a total cystectomy of the hydatid cysts, almost 100 cysts with multiple peritoneal and parietal locations, one of which was fistulized in the skin, associated with a total splenectomy, retrograde appendectomy and a disconnection of the cholecysto-duodenal fistula with duodenal closure and a retrograde cholecystectomy associated with a choledecotomy with extraction of 3 stones at the level of the choledochus and drainage of the VBP by Kehr drain. The postoperative course was simple and the patient was discharged on the sixth day with an adjuvant treatment with albendazole for three months.

Through this observation and in the light of the data in the literature, we were able to insist in our present work on the diagnostic difficulties generated by this unusual location of the hydatid cyst as well as the considerable contribution of imaging (CT++) allowing both a positive and very precise topographic diagnosis. We were also able to focus on surgical treatment as an indispensable pillar of the management of this disease as well as the increasingly fundamental role of medical treatment, particularly in the prevention of recurrences.

**Conclusion:**

Peritoneal hydatidosis is a rare but serious complication of hydatid disease.

The positive diagnosis is based on epidemiological, clinical, and paraclinical arguments represented essentially by CT scan.

Early diagnosis and treatment of primary sites as well as optimal surgical management of peritoneal hydatidosis determine the prognosis.

## Highlights

• Peritoneal hydatidosis is a parasitic condition secondary to seeding of the peritoneal serosa by Echinococcus granulosus larvae.
• It is often secondary to rupture or fissuring of hepatic hydatid cysts. It's a rare parasitic condition.
• Its diagnosis is based essentially on biological and especially radiological exploration techniques, it's treatment remains primarily surgical, Antiparasitic medical treatment is prescribed to prevent recurrence.
• The prognosis is generally good and the evolution is essentially marked by the risk of recurrence, especially in disseminated and late forms.

## 1 Introduction

Peritoneal hydatidosis (PH) is a parasitic condition secondary to seeding of the peritoneal serosa by Echinococcus granulosus larvae. It is often secondary to rupture or fissuring of hepatic hydatid cysts.

It is a rare parasitic condition. It represents about 5–16% of all hydatid cyst locations [1].

Its diagnosis is based essentially on biological and especially radiological exploration techniques [2]. Its treatment remains primarily surgical. Antiparasitic medical treatment is prescribed to prevent recurrence [3,4].

The prognosis is generally good and the evolution is essentially marked by the risk of recurrence, especially in disseminated and late forms [5].

We report a case of peritoneal hydatidosis of a particular form with more than 100 abdomino-pelvic hydatid cysts with multiple hepatic, peritoneal, parietal, splenic, and pelvic localizations in the department of visceral surgery 35 of the Ibn Rohd Hospital in Casa.

### 1.1 Patient and observation

She is Mrs xxx 29 years old, married and mother of 2 children. Admitted to the Visceral Surgical Emergency Department 35 of the IBN ROCHD Hospital for the management of a disseminated peritoneal hydatidosis. No particular pathological history.

The clinical examination revealed a soft abdomen with an epigastric crust.

The physical examination, and the rest of the somatic examination was unremarkable.

The biological workup showed a slightly disturbed liver function test and a strongly positive hydatid serology.

An abdominal ultrasound revealed: a reshaped hydatid cyst of segment III of the liver fistulized in the subcutaneous soft tissues measuring 4.19*1.98 cm and contunited with another formation measuring 3*2 cm.

Abdominal and pelvic CT scan in axial and sagittal section showed multiple hepatic, splenic and peritoneal cystic formations of which one of the hepatic lesions is fistulized to the wall of which the most voluminous is pelvic measuring 40*43mm and a cystic umbilical formation measuring 13*10mm with main biliary tract seat of a dense material with upstream dilatation of 10mm hydatid origin with the presence of an aerobilic that can evoke a bilio-digestive fistula, the pre-anaesthetic assessment was correct.

**Fig. 1:**
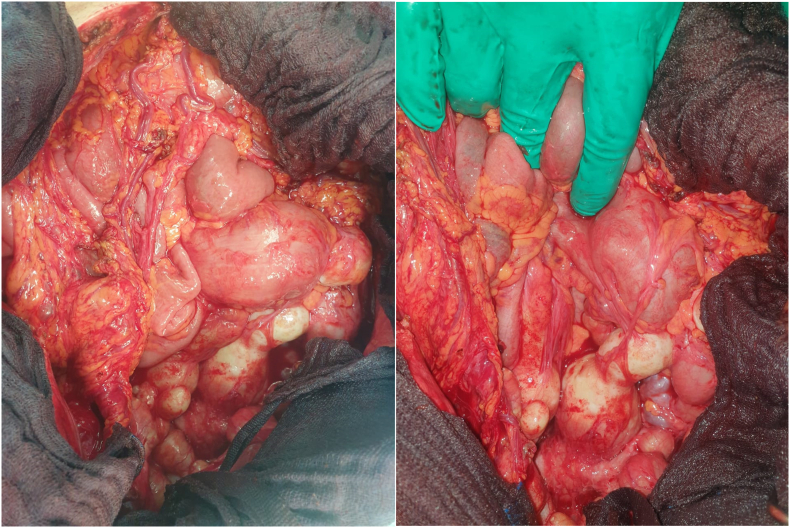
Intraoperative view showing.

A surgical operation was programmed with the presence of several abdomino-pelvic hydatid cysts which were distributed as follows 70 peritoneal cysts, 18 cysts of parietal localization of which one was fistulized to the skin, 12 pelvic cysts with a multi-cystic spleen (Fig. 1), we also note the adhesion of the peritoneal cysts to the appendix with the presence of a cholecysto-duodenal fistula and an atrophic gall bladder, main biliary tract of 15 mm with 3 lithiasis.

**Fig. 2:**
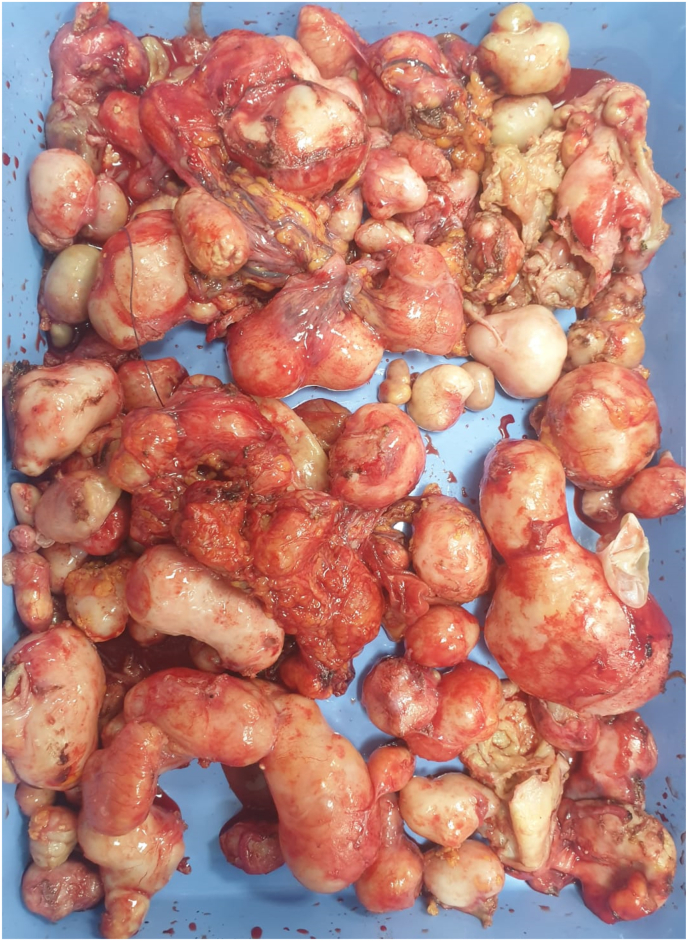
After operative view showing.

✓ For the peritoneal cysts, they were small with a weight varying between 10 gramsand 45 g
✓ for the parietal cysts, 17 cysts were estimated to weigh 50 g each with the largestbeing fistulised to the skin with an estimated weight of 100 g
✓ for 12 pelvic cysts, they were small with a weight ranging from 15 g to 70 g
✓ for one multi-cystic spleen, its weight was estimated at 350 g
  ❖ with a total weight of all cysts estimated at 1kg 360g (Fig. 2)

The surgical procedure consisted of a total cystectomy of the hydatid cysts associated with a total splenectomy, retrograde appendectomy and a disconnection of the cholecysto-duodenal fistula with duodenal closure and a retrograde cholecystectomy associated with a choledecotomy with extraction of 3 calculi at the level of the choledochus and draining of the VBP by Kehr drainthe surgical procedure was performed on a scheduled date with a correct pre-anesthetic assessment; the procedure was performed by an assistant professor in general surgery and two residents in the same specialty

The surgical procedure was performed on a scheduled date with a correct pre-anesthetic assessment; the procedure was performed by an assistant professor in general surgery and two residents in the same specialty.

The operation was performed in the operating room of the P35 visceral emergency department at the CHU ibn rochd hospital in Casablanca, Morocco the patient was satisfied with the intervention and the improvement of his health in the short and long term.

The postoperative evolution was without particularity, with the patient leaving the hospital at 6 days post-op. patient follow-up was every 15 days with a complete clinical examination with primary care of the surgical wound with instructions to change the dressing every 2 days and take antibiotics, anticoagulants and antlagics for 15 days and to avoid carrying heavy loads until complete healing then the patient was hospitalized after 6 months for residual calculus at the level of the VBP where she benefited from an ERCP (endoscopic retrograde cholangiopancreatography)

This case report is compliant with the SCARE Guidelines 2020 [20].

## 2 Discussion

Peritoneal hydatidosis is a rare but serious complication of hydatid disease [[1], [2], [3]]. It represents 5–16% of all localizations combined according to European series [4].

In the literature, the age distribution of hydatidosis generally shows a predominance of the disease in young adults [7,8].

Peritoneal echinococcosis is most often secondary to rupture or fissuring of a KH of hepatic origin in 66–85% of cases or of splenic origin in 10–20% of cases [1,5].

Primary peritoneal hydatidosis is exceptional. The rupture of the primary cyst is spontaneous in 78% of cases [6]. It is favored by the superficial location of the cyst, its large size, thin wall and high intracystic pressure. Traumatic rupture is most often iatrogenic during surgery or a diagnostic procedure such as liver biopsy or transhepatic cholangiography, and may also be secondary to abdominal contusion or a traffic accident.

The stages that follow the rupture reflect the reaction of the peritoneal serosa to the hydatid aggression. They present as simple hydatid ascites when the cyst is monolocular, free hydatids in the large peritoneal cavity when the cyst is multivesicular, or hydatid peritonitis if the cystic content is infected.

In the absence of treatment, these early forms can evolve in two ways: either subserosal grafting with development of peritoneal cysts is by far the most frequent, it can be either a multiple generalized form with sometimes more than 100 cysts as in the case of our patient or a form localized to a region of the abdomen. It can be a pseudo-tubercular form with a cluster of multiple punctiform cysts reminiscent of the miliary granulations of tuberculous peritonitis. The nature of the infecting material remains controversial. FERRAND and BARSOTTI [7] attribute this seeding to the projection of scolexes and daughter vesicles, whereas BENEX [8] affirms that only the germinal membrane is at the origin of the peritoneal infestation.

Encysted collections are characterized by the existence of a neoformed encystment membrane isolating the ruptured cystic content from the rest of the peritoneal cavity.

Acute intraperitoneal rupture is often discovered per operatively during emergency laparotomy [6].

However, the association of an acute abdominal picture with signs of allergy (urticaria, shock, etc.) should attract attention, especially in the presence of the notion of trauma.

The classic forms, late and insidious, have a polymorphic symptomatology. This can easily be explained by the diversity of cyst locations [13].

The functional signs are dominated by pain and signs of compression (jaundice, PH, urinary and rectal signs …) which represented in our patients the clinical translation of the very advanced forms. The general state remains preserved for a long time.

Medical imaging is an essential step in the diagnosis of peritoneal hydatidosis. Imaging techniques are nowadays numerous and efficient and the images obtained are often highly suggestive. They allow to establish the positive diagnosis of hydatid cysts, to confirm their location, to contribute to therapeutic strategies and to ensure the monitoring of treated subjects.

Ultrasound is considered to be the first-line examination for the diagnosis and detection of hydatid disease in its abdominal locations, with a reliability of more than 90% in most series reported in the literature. As far as peritoneal hydatidosis is concerned, it has been proven that ultrasound demonstrates the primary Hydatid Cyst in case of secondary peritoneal hydatidosis, and facilitates the diagnosis of single or multiple primary peritoneal hydatid localizations in case of isolated peritoneal hydatidosis [9].

Ultrasound has other advantages. Indeed, it allows a study of the relationship of the hydatid cyst with the portal bifurcation, the suprahepatic veins, the inferior vena cava and the upper urinary tract in search of possible compression.

In abdominal hydatidosis, CT has completely revolutionized lesion and topographic diagnostic management, as well as therapy [10].

Since its advent, it has become an indispensable complementary examination as soon as a surgical decision is indicated. CT not only confirms the disease but also provides a very useful lesion map for the surgeon.

Abdominal CT (in spontaneous contrast and/or with intravenous iodine injection) allows an easy and much more precise diagnosis than ultrasound of the hydatid cyst, particularly in its peritoneal location [8,10,12]. CT not only allows the disease to be confirmed but is also able to map the different locations, which is extremely useful for the surgeon. Magnetic resonance imaging.

(MRI) easily allows the diagnosis of rupture, but also to monitor the evolution of the hydatid cyst under medical treatment [[11], [12], [13], [14], [15]].

The differential diagnosis can be made with other cystic or pseudocystic masses of the peritoneum: tuberculosis, gelatinous disease, serous cyst and cystic lymphangioma. Hydatid serology is therefore of great interest; however, it is a good means of monitoring after treatment.

The treatment of peritoneal hydatidosis remains above all surgical [14,16]. The aim is to treat the peritoneal cysts and the primary hydatid cyst at the same time [10]. It is associated with medical treatment, preoperatively to sterilize the cysts but especially postoperatively to avoid recurrences which are quite frequent in peritoneal hydatidosis [17,18], as one can never be sure of having completely removed the hydatid cysts.

In acute forms, the treatment consists of evacuating the peritoneal effusion (hydatid sand, daughter vesicles, bile, blood) and cure the infesting viscera. A meticulous peritoneal cleansing completes the operation.

In late forms, the peritoneal time may require the association of several procedures depending on the topography of the lesions and the importance of the dissemination. However, certain rules must be borne in mind: always free the pelvis first, otherwise pelvic compression may occur, the prognosis of which may be vital; adapt the duration of each operative stage to the resistance of the patient, starting with the destruction of the largest cysts or those that compress the intestine, and evacuate the primary hydatid cysts from the first operative stage. A complete cure in a single operation is desirable [17,18]. It is recommended to perform excision (cystectomy, peri-cystectomy) of cysts that are easily and safely accessible, and partial cystectomy (resection of the protruding dome) with evacuation of the parasite for deep cysts in close contact with the vessels, mesos and viscera [12], when local and general conditions do not allow it: multioperated patient with dense adhesions making dissection laborious and hemorrhagic we recommend “puncture - aspiration - sterilization” of cysts. Heavy procedures, such as pancreatectomies or major hepatectomies are not justified in this benign pathology, given their high risk of mortality and morbidity. In highly disseminated forms, such as hydatid pseudotuberculosis of the peritoneum, with thousands of small pinhead cysts, it is understandable that the surgeon is unable to provide any help to the patient [17,18],

The prognosis of the disease depends on the extent of peritoneal dissemination, the existence of visceral localizations and their severity, the general condition of the patient and the number of operations he/she has already undergone, the completeness of the cure and the experience of the surgeon.

Long term monitoring is essentially aimed at detecting recurrences requiring reoperations. Since one can never be sure of having performed a complete and exhaustive removal of the lesions, any patient operated on for peritoneal hydatidosis must therefore be monitored as regularly and closely as possible, from the immediate postoperative period until several years later [19]. This monitoring is essentially based on the following controls: Clinical, always delayed in relation to imaging. Serological: the addition of the results of several tests is necessary due to the low sensitivity and specificity of the latter. With medical imaging: mainly ultrasound, CT has a much more considerable but expensive contribution.

## 3 Conclusion

Peritoneal hydatidosis is a rare but serious complication of hydatid disease.

The positive diagnosis is based on epidemiological, clinical, and paraclinical arguments represented essentially by CT scan.

Surgery is the mainstay of treatment of peritoneal hydatidosis, based on radical or conservative methods which must act on both the peritoneal hydatidosis and the primary visceral hydatid cyst. Medical treatment also has its place in this condition. It represents an interesting therapeutic alternative for inoperable patients and for disseminated and multivisceral hydatidosis. It is also of interest in the prevention of relapses and recurrences by providing a framework for the surgical intervention.

Long-term postoperative surveillance is necessary to detect any recurrence. It is essentially based on ultrasound/CT and serology.

Early diagnosis and treatment of primary sites as well as optimal surgical management of peritoneal hydatidosis determine the prognosis.

## Ethical approval

I declare on my honor that the ethical approval has been exempted by my establishment.

## Sources of funding

None.

## Authors contribution

El Bakouri Abdelilah: writing the paper and operating surgeon.

Fatine Amine: writing the paper and operating surgeon.

Eddaoudi Yassine: Corresponding author writing the paper and operating surgeon.

Bouali Mounir: study concept.

El Hattabi Khalid: study concept.

Bensardi Fatimazahra: study concept.

Fadil Abdelaziz: correction of the paper.

## Registration of research studies

Researchregistry2464.

## Consent

Written informed consent for publication of their clinical details and/or clinical images was obtained from the patient.

## Guarantor

Dr Fatin Amin.

## Provenance and peer review

Not commissioned, externally peer-reviewed.

## Declaration of competing interest

The authors declare having no conflicts of interest for this article.
